# Loop-mediated isothermal amplification (LAMP) test for diagnosis of uncomplicated malaria in endemic areas: a meta-analysis of diagnostic test accuracy

**DOI:** 10.1186/s12936-020-03283-9

**Published:** 2020-06-19

**Authors:** Denesh Selvarajah, Cho Naing, Norah Htet Htet, Joon Wah Mak

**Affiliations:** 1 School of Medicine, International Medical University, Kuala Lumpur, 5700 Malaysia; 2 Institute for Research, Development and Innovation (IRDI), International Medical University, Kuala Lumpur, Malaysia; 3 Faculty of Tropical Heath and Medicine, James Cook University, Queensland, Australia

**Keywords:** Malaria, Diagnostic tests, Accuracy, Assays, Meta-analysis

## Abstract

**Background:**

The global malaria decline has stalled and only a few countries are pushing towards pre-elimination. The aim of the malaria elimination phase is interruption of local transmission of a specified malaria parasite in a defined geographical area. New and improved screening tools and strategies are required for detection and management of very low-density parasitaemia in the field. The objective of this study was to synthesize evidence on the diagnostic accuracy of loop-mediated isothermal amplification (LAMP) test for the detection of malaria parasites among people living in endemic areas.

**Methods:**

This study adhered to the Preferred Reporting Items for Systematic Reviews and Meta-Analysis for Diagnostic Test Accuracy (PRISMA-DTA) guideline. Relevant studies in the health-related electronic databases were searched. According to the criteria set for this study, eligible studies were identified. The quality of included studies was evaluated with the use of a quality assessment checklist. A summary performance estimates such as pooled sensitivity and specificity were stratified by type of LAMP. Bivariate model for data analyses was applied. Summary receiver operating characteristics plots were created to display the results of individual studies in a receiver operating characteristics space. Meta-regression analysis was performed to investigate the sources of heterogeneity among individual studies.

**Results:**

Twenty-seven studies across 17 endemic countries were identified. The vast majority of studies were with unclear risk of bias in the selection of index test. Overall, the pooled test performances were high for *Pan* LAMP (sensitivity: 0.95, 95% CI 0.91 to 0.97; specificity: 0.98, 95% CI 0.95 to 0.99), *Plasmodium falciparum* (*Pf*) LAMP (sensitivity: 0.96, 95% CI 0.94 to 0.98; specificity: 0.99, 95% CI 0.96 to 1.00) or for *Plasmodium vivax* (*Pv*) LAMP from 6 studies (sensitivity: 0.98, 95% CI 0.92 to 0.99; specificity: 0.99, 95% CI 0.72 to 1.00). The area under the curve for Pan LAMP (0.99, 95% CI 0.98–1.00), *Pf* LAMP (0.99, 95% CI 0.97–0.99) and *Pv* LAMP was (1.00, 95% CI 0.98–1.00) indicated that the diagnostic performance of these tests were within the excellent accuracy range. Meta-regression analysis showed that sample size had the greatest impact on test performance, among other factors.

**Conclusions:**

The current findings suggest that LAMP-based assays are appropriate for detecting low-level malaria parasite infections in the field and would become valuable tools for malaria control and elimination programmes. Future well-designed larger sample studies on LAMP assessment in passive and active malaria surveillances that use PCR as the reference standard and provide sufficient data to construct 2 × 2 diagnostic table are needed.

## Background

The global malaria decline has stalled and only a few countries are pushing towards pre-elimination [1]. The aim of the malaria elimination phase is interruption of local transmission of a specified malaria parasite in a defined geographical area [2]. Although there were an estimated 20 million fewer malaria cases in 2017 than in 2010, the World Malaria Report 2018 highlights that no significant progress in reducing global malaria cases was made in the period 2015–2017 [1]. For instance, the ten highest malaria burden African countries had an estimated 3.5 million more malaria cases in 2017 compared with the previous year [1]. In order to reduce and eventually eliminate the parasite reservoir, early detection of infected individuals and effective treatment with gametocidal drugs are important, whilst mosquito activities are minimal [1, 2].

To meet the target of malaria elimination, surveillance for submicroscopic infections is crucially important. Strategies to interrupt malaria transmission include prompt identification and treatment of asymptomatic infections. The majority of asymptomatic had low parasite densities, undetectable by microscopy or RDT, but can currently be identified reliably by PCR. However, PCR is time and resource intensive, and it is not a viable method in field operations. New and improved screening tools and strategies are required for detection and management of very low-density parasitaemia in the field [1, 2].

The diagnostic methods currently used for mass screening with microscopy or on-site rapid diagnostic tests (RDTs) are not sensitive enough to detect low-density malarial infections [3]. The suitability of RDTs for surveillance of malaria in low transmission settings with low density and sub-microscopic infections is a concern. The use of polymerase chain reaction (PCR) for case detection could yield higher sensitivity to detect even a single parasite in a blood sample (approximately 10 to 30 µl of blood volume) [4, 5]. However, PCR is expensive and requires thermocycling conditions, which is impracticable in the field setting, especially in countries with limited resources. Loop-mediated isothermal amplification (LAMP) theoretically enables the detection of low density and sub-microscopic infections with better accuracy and greater ease [6–8]. In brief, LAMP is a molecular technique for nucleic acid amplification and performed to determine the presence of *Plasmodium* parasites in the blood samples based on the presence or absence of *Plasmodium* DNA. Primer sequences for LAMP amplification of the *Plasmodium* genus are for detection of *Plasmodium falciparum, Plasmodium vivax, Plasmodium ovale* and *Plasmodium malariae.*

Studies using LAMP for the detection of malaria in endemic areas are available. However, performance results reported in these studies are inconsistent. Until now, a comprehensive and systematic review of studies addressing the diagnostic accuracy of LAMP test in detection of human malaria is limited. The objective of the present study was to synthesize evidence on the diagnostic accuracy of LAMP test for the detection of malaria parasites among people living in endemic areas.

## Methods

This study was performed, according to the Preferred Reporting Items for Systematic Reviews and Meta-Analysis for Diagnostic Test Accuracy (PRISMA-DTA) guidelines [9] (Additional file 1).

### Search strategy

Electronic databases of Medline, EMBASE, Web of Science, Cochrane systematic review database, the Latin American and Caribbean Health Sciences Literature (LILACS) and African Journals Online were searched for relevant studies published in English until May 2019 and an updated search in March 2020. The search was conducted using keywords and Boolean operators: (“malaria” OR “plasmodium”) AND (“LAMP” OR “loop-mediated” “dipsticks” OR “RDT” OR “rapid diagnosis” OR “rapid onsite diagnosis” OR “ICT” OR “immunochromatographic”) OR (“microscopy” OR “PCR”). The references of retrieved articles and relevant reviews manually were checked for any additional studies.

### Study selection

For selection of eligible studies, the criteria were set as described below.

Type of studies: Any study design, if it had evaluated the accuracy of LAMP in detection of malaria.

Participants: Participants living in the malaria endemic countries.

Index test: Any type of LAMP for diagnosis of malaria.

Comparator test: No comparator or an alternative diagnostic test (e.g. RDT/microscopy).

Target conditions: Detection of human malaria cases, regardless of parasite species.

Reference standard: PCR

Outcomes: The main outcome was measured in terms of sensitivity and the specificity of the diagnostic test of interest. Sensitivity refers to the probability that the index test result will be positive in an infected case. Specificity refers to the probability that the index test result will be negative in a non-infected case [10, 11].

To be eligible, a study must have provided sufficient data to construct 2 × 2 tables (true positive, false positive, false negative, true negative).

### Exclusion criteria

Studies were excluded, if they used LAMP for detection of other settings or other disease apart from malaria (e.g. malaria in non-endemic areas, tuberculosis). Studies on special groups such as pregnant women or travellers were not included. Studies without sufficient data to construct 2 × 2 tables were not considered.

### Data extraction and management

One review author (DS) screened title and abstracts on the basis of the inclusion criteria. The same review author extracted information from all included studies. Data extracted were first author, publication year, country, setting, characteristic of study (sample size, details of tests used), characteristic study participants and outcome data. Information collected were cross-checked by another review author (NHH). Any discrepancy between the two investigators was resolved by discussion and consensus.

### Methodological quality assessment

To evaluate the methodological quality of included studies, the Quality Assessment of Diagnostic Accuracy Studies-2 (QUADAS-2) checklist was used. As described elsewhere [12], the QUADAS-2 checklist has four standard domains (‘patient selection’, ‘index tests’, ‘reference standards’ and ‘flow and timing’). The checklist consists of signalling questions under each domain and the answers for these signalling questions allow the assessment of the risk of bias for each domain.

Statistical heterogeneity between the studies was measured as *I*^2^ values, which describes the proportion of total variation in study estimates due to heterogeneity; *I*^2^ values > 50% is regarded as substantial heterogeneity [10, 13]. The pooling of data was done only when there were two or more studies that used a particular type of LAMP that targeted the same species/genus.

As described elsewhere [11], sensitivity and specificity for each included study were described in the forest plots. A summary performance estimate was stratified by type of LAMP. Bivariate model for data analyses was used. Summary receiver operating characteristics (SROC) plots were created to display the results of individual studies in a receiver operating characteristics (ROC) space. This provides information on the overall performance of a test across different thresholds. The best diagnostic test is positioned in the top left hand corner of the ROC space, whereby both the sensitivity and specificity are close to 1.0 [10]. The area under the curve (AUC) shows the analytic summary of the diagnostic test performance among the included studies. An AUC of 0.97 or above demonstrates excellent accuracy [14]. Meta-regression analysis was performed to investigate the sources of heterogeneity among individual studies. Covariates such as sample size, study design, and the blinding of the index test and reference test results were used for the meta-regression analysis. A *p *< 0.05 in the joint model was considered to contribute to heterogeneity. The potential publication bias was assessed by inspection of a funnel plot [15]. All statistical analyses were done with *midas* package in STATA 15.0 and RevMan 5.3 (The Nordic Cochrane Centre).

## Results

**Fig. 1:**
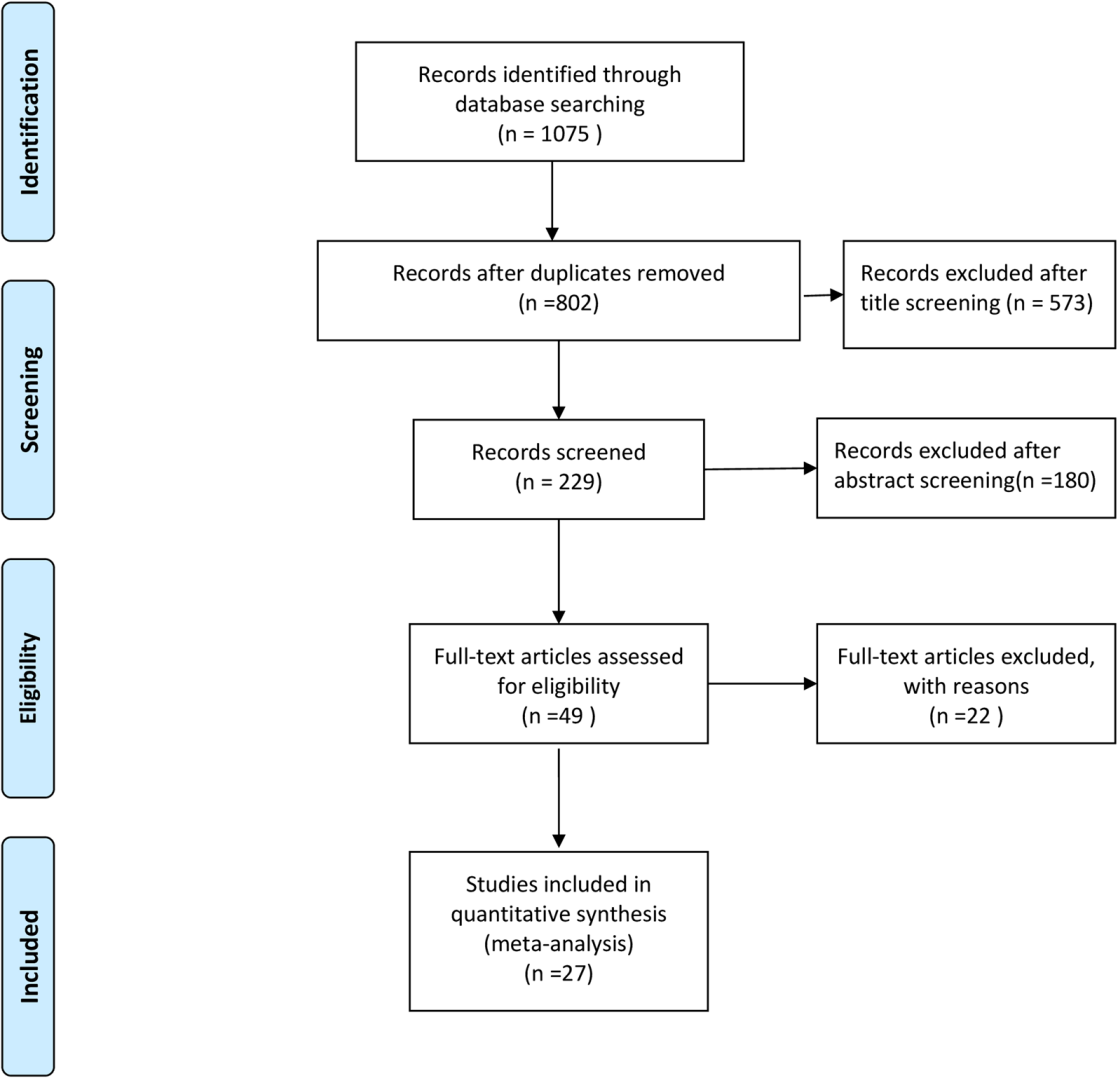
Study selection process

Figure 1 illustrates the four-phase study selection process. The initial search in the electronic databases yielded 1075 citations. After removal of duplicates and by title and abstract screening, a total of 49 articles were eligible for full-text screening. Finally, 27 articles (9769 participants) were selected for the current meta-analysis. The reasons of exclusion of 22 studies were summarized (Additional file 2).

### Characteristics of the included studies

The characteristics of studies included in the current analysis are presented (Additional file 3). Of these 27 studies, more than half of the studies included were cross-sectional design (54%, 14/27), while the remaining 13 studies were case–control designs. A subset of 15 studies (19 data sets) assessed Pan LAMP [6, 8, 16–28], while 14 studies (17 data sets) used *Plasmodium falciparum* (*Pf*) LAMP [7, 8, 18, 20, 23, 25, 27–34] and only 6 studies (6 data sets) used *Plasmodium vivax* (*Pv*) LAMP [27, 28, 32, 35–37].

**Fig. 2:**
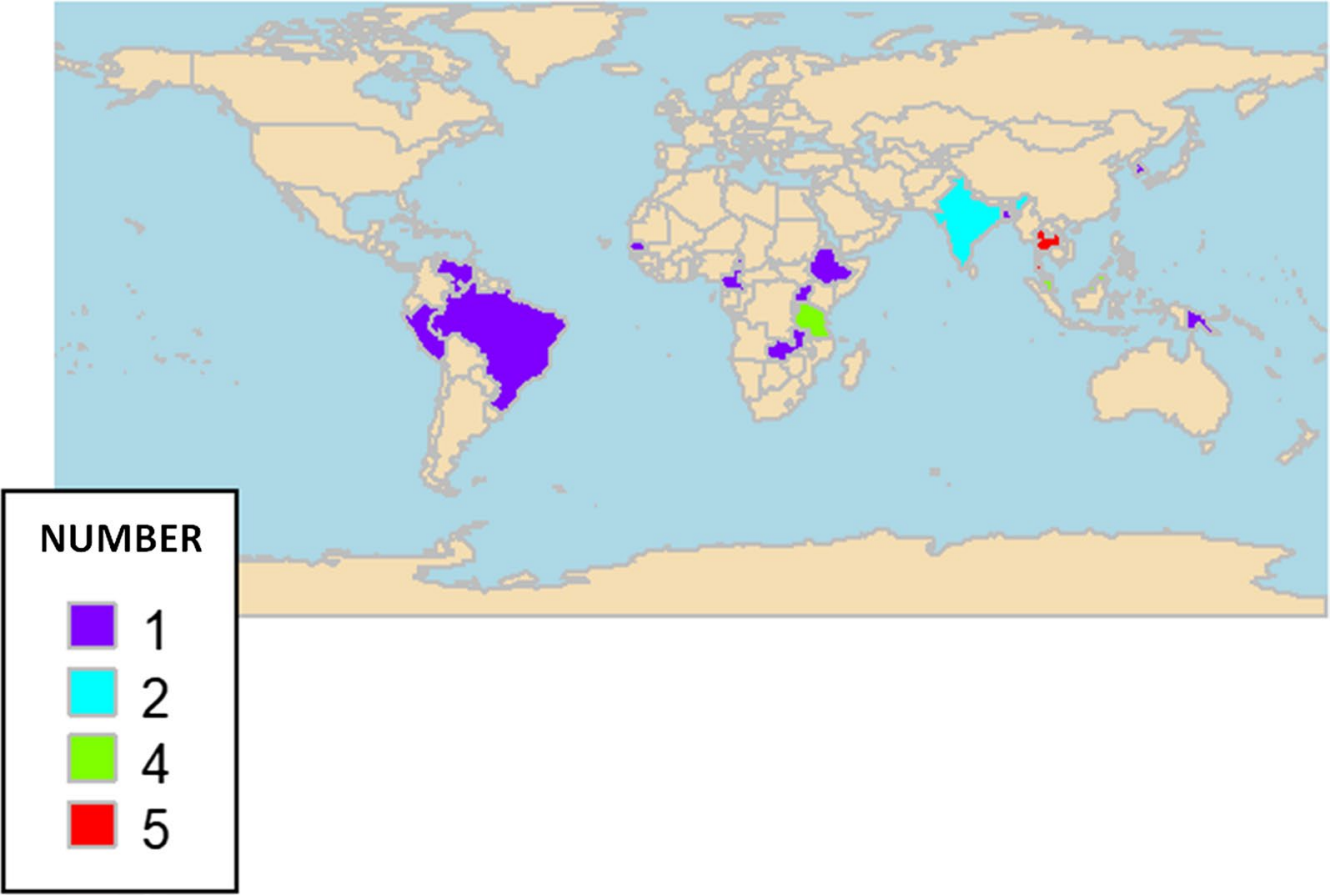
Geographical distribution of the included studies

Of these, two studies (7.4%) were multicountry studies that were carried out in India and Thailand [19] or Gambia, Papua New Guinea and Malaysia [20]. Of the remaining 25 single studies, the majority were done in the Asian region [7, 19, 20, 23, 25, 27, 29–32, 36–39], while ten studies in African countries [6, 8, 16–18, 21, 22, 24, 33, 34], and three studies in the South American countries Brazil [28], Peru [26] and Venezuela [35]. Figure 2 shows the global distribution of the 27 included studies. The number of participants ranged widely from a minimum of 35 [8] to a maximum of 3008 [24]. The publication years covered from 2006 to 2019, and the majority were published between 2015 and 2019 [6, 8, 20–28, 33, 34, 37, 39].

### Methodological quality of the included studies

**Fig. 3:**
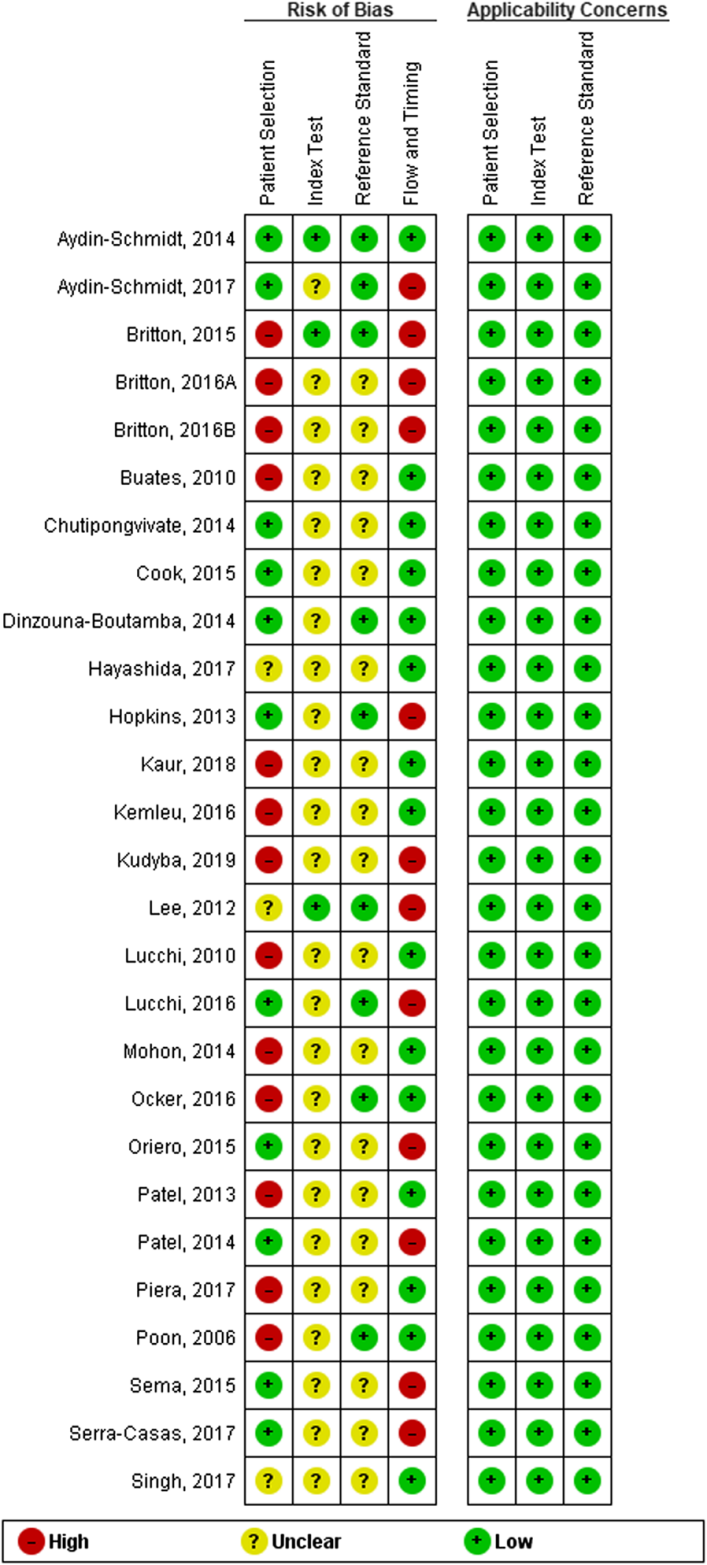
Methodological quality assessment of each individual study

The methodological quality of individual study is provided in Fig. 3. Many studies included in the current analysis were with high or unsure risk of bias. The summary of the methodological quality assessment across all studies are in Additional file 4. Less than half of the studies had either high risk of bias (48%) or low risk of bias (41%) in patient recruitments. The majority were with unclear risk of bias in the selection of index test (89%) or the reference standard (67%). There were low concerns on the ‘applicability’ of the included studies with regard to patient selection (i.e. low concern because the included patients were matched the targeted population), index test (i.e. low concerns because the conducts or interpretation of LAMP is different from the designated procedures) and reference standard (i.e. low concerns because the reference standard PCR is useful for detection of malaria).

### Test performances

**Fig. 4:**
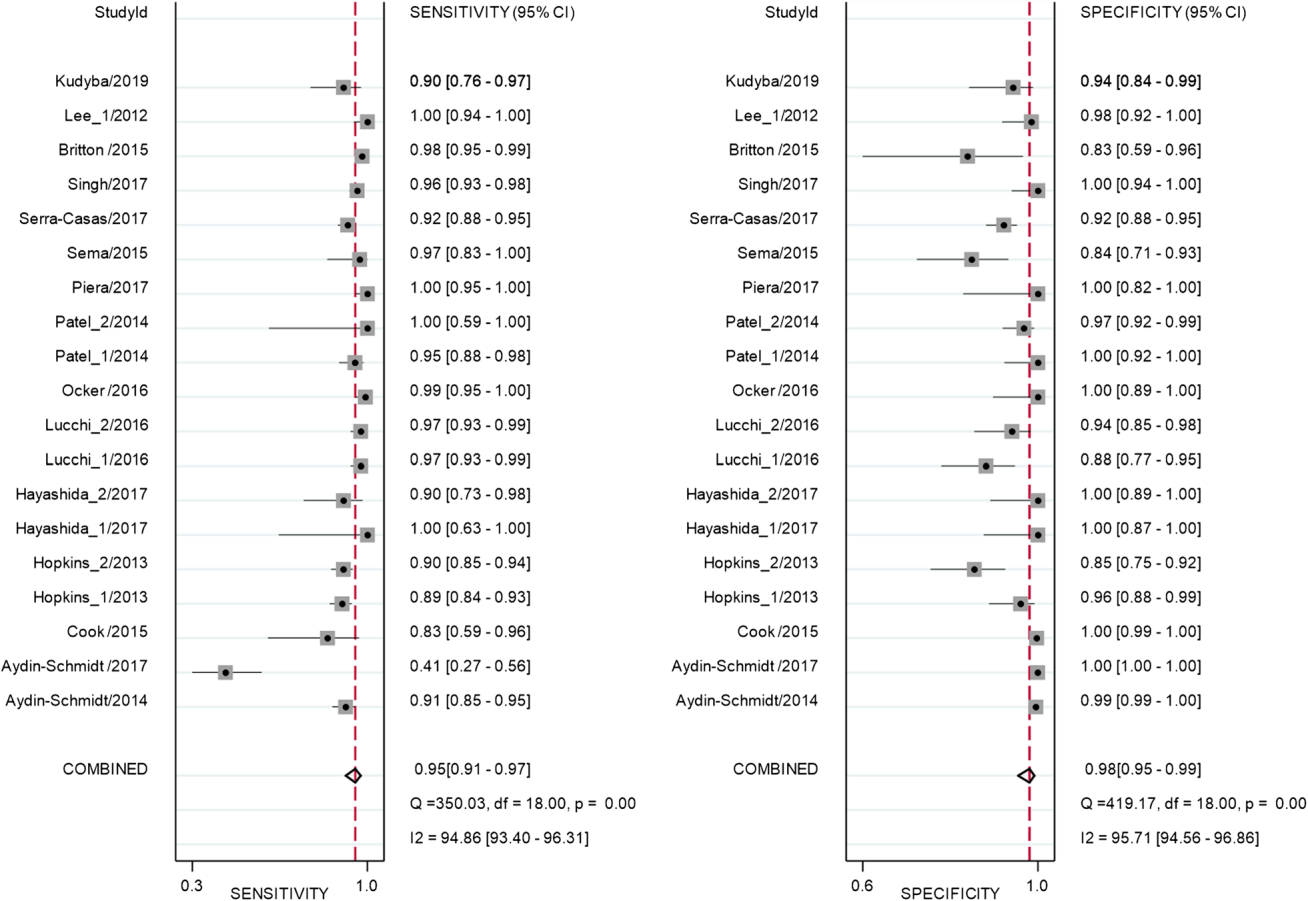
Forest plot of sensitivity and specificity for Pan LAMP

Test performance of the studies that used Pan LAMP is provided in Additional file 5. Overall, the pooled sensitivity and specificity from 15 studies with 19 datasets that used Pan LAMP for detection of malaria were high at 0.95 (95% CI 0.91 to 0.97) and 0.98 (95% CI 0.95 to 0.99), respectively (Fig. 4).

**Fig. 5:**
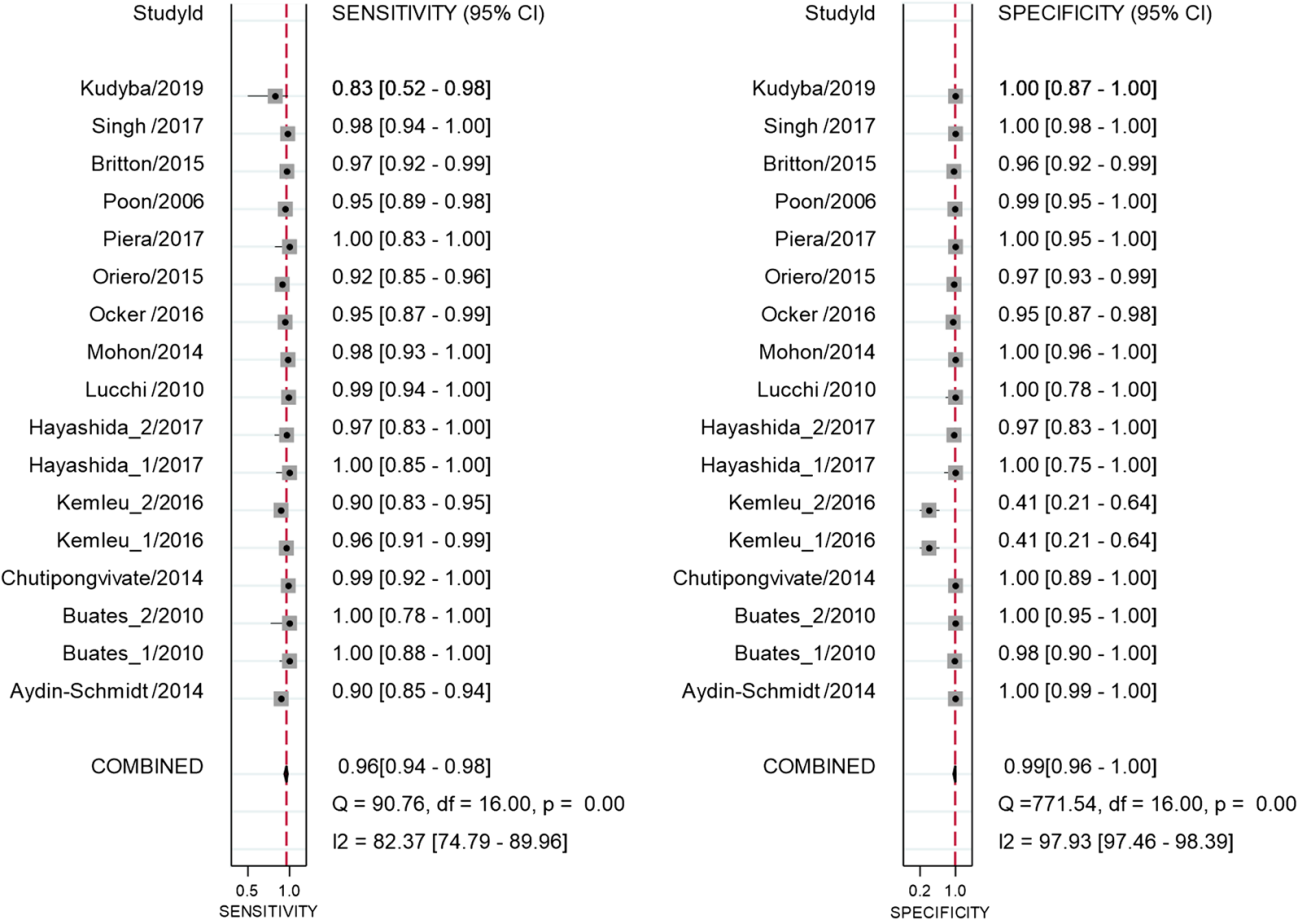
Forest plot of sensitivity and specificity for *Pf* LAMP

**Fig. 6:**
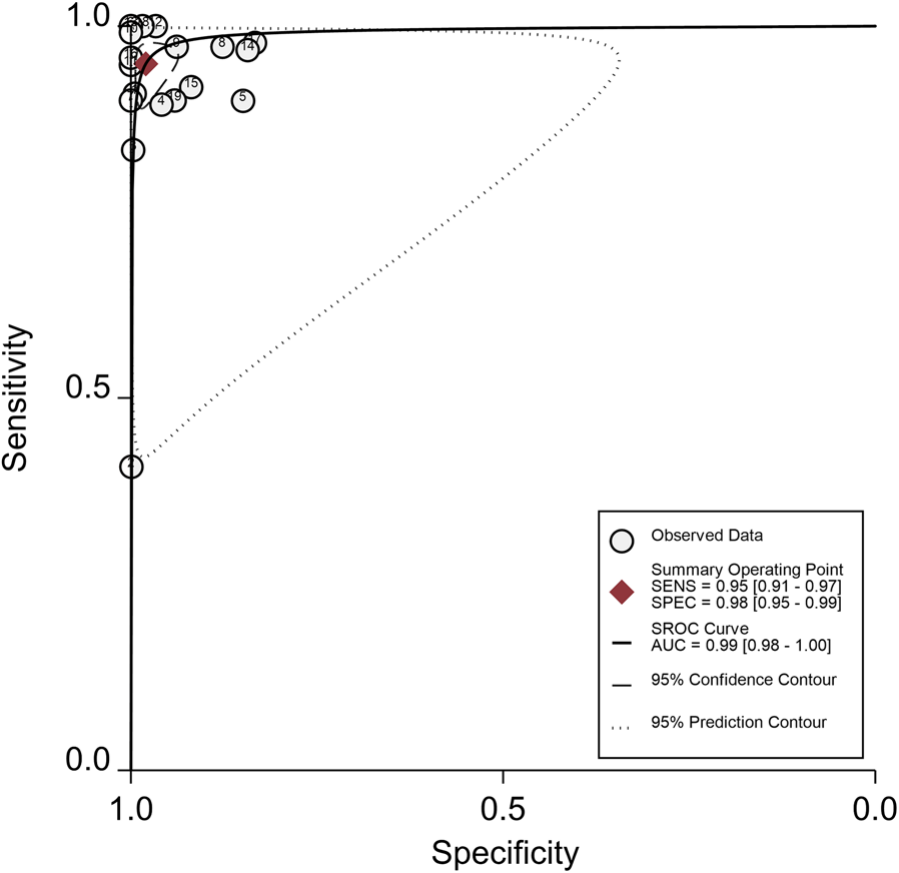
SROC plot of studies that used Pan LAMP

Overall, there were high pooled sensitivity from 14 studies with 17 datasets (0.96, 95% CI 0.94 to 0.98) and specificity (0.99, 95% CI 0.96 to 1.00) for the *Pf* LAMP (Fig. 5). The *Pv* LAMP from 6 studies showed (sensitivity: 0.96, 95% CI 0.91 to 0.99) and 0.99 (specificity: 95% CI 0.56 to 1.00) (Additional file 6). An SROC model for the Pan LAMP is shown in Fig. 6. The AUCs for Pan LAMP *(*0.99, 95% CI 0.98–1.00), the *Pf* LAMP (0.99, 95% CI 0.96–1.0) and the *Pv* LAMP was (1.0 95% CI 0.98–1.0) indicated that the diagnostic performance of these tests were within the excellent accuracy range (Additional files 7 and 8).

There was substantial between-study heterogeneity as the *I*^2^ values for sensitivity and specificity of all LAMP tested were 94.86% and 95.71%, respectively (Figs. 4, 5). To investigate the source of heterogeneity, meta-regression analysis was performed with four covariates such as sample size, blinding for index test, blinding for reference test and the study design. The results of meta-regression are presented in Table 1. Of these potential confounding factors, sample size had the greatest impact on the sensitivity and specificity of Pan LAMP.

**Table 1:** Meta-regression output of sensitivity and specificity of Pan LAMP

Description	Number of datasets^a^	Sensitivity (95% CI)	p1	Specificity	p2
Sample size	19	0.94 (0.90–0.96)	1.00	0.97 (0.94–0.99)	1.00
Blinding for reference test	19	0.84 (0.58–0.95)	0.13	0.98 (0.86–1.00)	0.98
Blinding for index test	19	0.88 (0.46–0.98)	0.52	1.00 (0.97–1.00)	0.11
Study design	19	0.99 (0.94–1.00)	0.06	0.98 (0.83–1.00)	0.91

*p1* p value for sensitivity, *p2* p value for specificity

^a^Single studies with more than 1 data set are included

## Discussion

The present review included 27 studies (9769 participants) across 17 malaria endemic countries in the African and South-East Asia regions. The major observations are as follows:

1. The pooled sensitivities and specificities of the Pan LAMP, the *Pf* LAMP and the *Pv* LAMP were high in terms of reference test PCR.
2. Species-specific LAMP tests (*Pf* LAMP, *Pv* LAMP) had higher levels of sensitivities and specificities than that of the genus-specific LAMP test (i.e. Pan LAMP).

The vast majority of the primary studies in the present analysis (92%) was conducted in the African and South-East Asia regions that reported ≈ 97% of the total malaria cases globally in the year 2016 [2]. This implied that the findings of primary studies represented the endemic areas targeted for malaria control/elimination. The current findings of sensitivities were comparable with a published meta-analysis, which compared the accuracy of LAMP with PCR [40]. However, the earlier meta-analysis showed a specificity of 91% [40], which was lower than the current findings. This variation might be due to the differences in the number of primary studies in these meta-analysis studies. The earlier analysis included only four primary studies, while the current analysis consisted of 27 studies. The current findings of high sensitivity of LAMP suggested that it would be a suitable test to ‘rule-out’ malaria, when the test shows a negative result. Moreover, the nearly perfect specificity of LAMP for detection of malaria suggests that this test would be a suitable test to ‘rule-in’ the disease, when it shows a positive result.

On stratification, the pooled sensitivities and specificities of species-specific LAMP tests (*Pf* LAMP, *Pv* LAMP) were higher than the genus-specific LAMP test (Pan LAMP). Pan LAMP is for initial screening of malaria, irrespective of speciation. This would help save costs through a prevention from over-treatment for malaria which is important in malaria endemic areas with limited resources.

Regarding the methodology, heterogeneity is expected to be substantial in diagnostic test accuracy (DTA) studies. Therefore, the models used in DTA reviews are by default random effects models [10] and this guidance was followed. Bivariate model, which recommended purely binary tests or when different studies reported similar thresholds was chosen [11]. The current report focused on sensitivity and specificity, rather than other accuracy measures. This is because any other measure would be calculated on the basis of these two main parameters.

### Study limitations

Due to small number of studies included in species-specific LAMPs, a low power to obtain the true accuracies was a concern. This concern was supported by a meta-regression analysis, showing that sample size in primary studies had an impact on the test performances of LAMPs. The majority of the included studies had unclear risk of bias because it was not clear whether the index test was interpreted without the knowledge of the results of the reference standard. Hence, there might be possibilities of over/under estimations of the test accuracies.

There are confounding factors that could have effects on the pooled sensitivities and specificities of the LAMP tests. For instance, a variation in types of PCR used in the primary studies (i.e. nested PCR (nPCR), multiplex PCR, real-time quantitative PCR (qPCR), reverse transcription PCR) might have different diagnostic accuracy in detection of malaria. It has been reported that nPCR had a lower specificity than qPCR for the diagnosis of malaria [40].

Immunity level of travellers from non-endemic countries is relatively low compared to the residences in endemic areas [41]. Immunity levels of pregnant mothers is also relatively lower than the non-pregnant participants probably due to physiological changes in pregnancy [42]. The current review did not include studies with travellers or pregnant women. Hence, the estimates resulted in our review were less likely of bias related to immune status.

Nevertheless, there are several features of LAMPs that make it a potential tool for field use in malaria control/elimination programmes; it has (i) a high specificity, which reduce the frequency of false-positive results [19], (ii) a high sensitivity, which could translate into a screening test with high PPV and NPV in areas of low malaria prevalence where attempts are being made to eliminate this disease [8], (iii) demonstrated efficacy in detecting and identifying even *P. vivax* infections, which often predominate in countries entering malaria elimination in Southeast Asia and Latin America [27, 35], (iv) majority of the LAMP assays described are less resource intensive than standard PCR tests [6, 7], and (v) it can be performed by technicians after appropriate training in a rural health clinic or field site [6].

## Conclusions

The current findings suggest that LAMP-based assays are appropriate for detecting low-level malaria parasite infections in the field and would become valuable tools for malaria control and elimination programmes. Future well-designed larger sample studies on LAMP assessment in passive and active malaria surveillances that use PCR as the reference standard and provide sufficient data to construct 2 × 2 diagnostic table are needed.

## Supplementary information

**Additional file 1.** PRISMA-DTA Checklist.

**Additional file 2.** Summary of excluded studies.

**Additional file 3.** Characteristics of included studies.

**Additional file 4.** Summary of the methodological quality assessment across all studies.

**Additional file 5.** Test performance of individual studies with Pan LAMP.

**Additional file 6.** Forest plot of sensitivity and specificity for *Pv* LAMP.

**Additional file 7.** SROC plot of studies that used *Pf* LAMP.

**Additional file 8.** SROC plot of studies that used *Pv* LAMP.

## Data Availability

All data generated or analysed during this study are included in this article and its additional information files.

## Abbreviations

AUC: Area under the curve
LAMP: Loop-mediated isothermal amplification
nPCR: Nested PCR
PRISMA-DTA: Preferred Reporting Items for Systematic Reviews and Meta-Analysis for Diagnostic Test Accuracy
PCR: Polymerase chain reaction
qPCR: Real-time quantitative PCR,
QUADAS-2: Quality Assessment of Diagnostic Accuracy Studies-2
RDT: Rapid on-site diagnostic test
ROC: Receiver operating characteristics
SROC: Summary receiver operating characteristics
