# Supplementary material for: Loop-mediated isothermal amplification (LAMP) test for diagnosis of uncomplicated malaria in endemic areas: a meta-analysis of diagnostic test accuracy
Source: Malar J. 2020 Jun 19;19:211. doi: 10.1186/s12936-020-03283-9 (PMC7305603; doi:10.1186/s12936-020-03283-9)
Supplement: Supplementary file 2 — Additional file 2. Summary of excluded studies. [file 12936_2020_3283_MOESM2_ESM.doc]

Additional File 2: Table S2. Summary of excluded studies.

| **No** | **Study** | **Reasons** | **Citations** |
| --- | --- | --- | --- |
| 1 | Lau, 2016 | PCR was not used as the reference test/standard | Lau Y, Jelip J, Lai M, Fong M, Mahmud R. Loop-mediated isothermal amplification assay for identification of five human Plasmodium species in Malaysia. Am J Trop Med Hyg. 2016;94(2):336-9. |
| 2 | Vallejo, 2015 | Insufficient data to construct 2x2 diagnostic table | Vallejo A, Martínez N, González I, Arévalo-Herrera M, Herrera S. Evaluation of the loop mediated isothermal DNA amplification (LAMP) kit for malaria diagnosis in *P. vivax* endemic settings of Colombia. PLoS Negl Trop Dis 2015;9(1):e3453. |
| 3 | Lau, 2011 | PCR was not used as the reference test/standard | Lau Y, Fong M, Mahmud R, Chang P, Palaeya V, Cheong F, et al. Specific, sensitive and rapid detection of human *Plasmodium knowlesi* infection by loop-mediated isothermal amplification (LAMP) in blood samples. Malaria J. 2011;10(1):197. |
| 4 | Oriero, 2015 | Some of the samples were not human samples | Oriero C, Van Geertruyden J, Jacobs J, D'Alessandro U, Nwakanma D. Validation of an apicoplast genome target for the detection of Plasmodium species using polymerase chain reaction and loop mediated isothermal amplification. Clin Microbiol Infect. 2015;21(7):686. |
| 5 | Poschl, 2010 | PCR was not used as the reference test/standard | Poschl B, Thekisoe O, Chutipongvivate S, Panagiotis K, Waneesorn J. Comparative diagnosis of malaria infections by microscopy, nested PCR, and LAMP in Northern Thailand. Am J Trop Med Hyg 2010;83(1):56-60. |
| 6 | Paris, 2007 | Insufficient data to construct 2x2 diagnostic table | Paris DH, Imwong M, Faiz AM, Hasan M, Yunus EB, Silamut K, et al. Loop-mediated isothermal PCR (LAMP) for the diagnosis of falciparum malaria. Am J Trop Med Hyg. 2007;77:972-6. |
| 7 | Han, 2007 | PCR was not used as the reference test/standard | Han E, Watanabe R, Sattabongkot J, Khuntirat B, Sirichaisinthop J, Iriko H, et al. Detection of four Plasmodium species by genus- and species-specific loop-mediated isothermal amplification for clinical diagnosis. J Clin Microbiol . 2007 ;45(8):2521-8 |
| 8 | Lu, 2011 | PCR was not used as the reference test/standard | Lu F, Gao Q, Zhou H, Cao J, Wang W, Lim C, et al. Molecular test for vivax malaria with loop-mediated isothermal amplification method in central China. Parasitol Res. 2011;110(6):2439-44. |
| 9 | Tao, 2011 | PCR was not used as the reference test/standard | Tao Z, Zhou H, Xia H, Xu S, Zhu H, Culleton R, et al. Adaptation of a visualized loop-mediated isothermal amplification technique for field detection of *Plasmodium vivax* infection. Parasit Vectors . 2011;4(1):115 |
| 10 | Yongkiettrakul, 2014 | Insufficient data to construct 2x2 diagnostic table | Yongkiettrakul S, Jaroenram W, Arunrut N, Chareanchim W, Pannengpetch S, Suebsing R, et al. Application of loop-mediated isothermal amplification assay combined with lateral flow dipstick for detection of *Plasmodium falciparum* and *Plasmodium vivax*. Parasitol Into. 2014;63(6):777-84 |
| 11 | Port, 2014 | Insufficient data to construct 2x2 diagnostic table | Port J, Nguetse C, Adukpo S, Velavan T. A reliable and rapid method for molecular detection of malarial parasites using microwave irradiation and loop mediated isothermal amplification. Malaria J. 2014;13(1):454 |
| 12 | Cuadros, 2015 | PCR was not used as the reference test/standard | Cuadros J, Perez-Tanoira R, Prieto-Perez L, Martin-Martin I, Berzosa P, Gonzalez V, et al. Field evaluation of malaria microscopy, rapid malaria tests and loop-mediated isothermal amplification in a rural hospital in South Western Ethiopia. PLoS One. 2015 ;10(11):e0142842 |
| 13 | Morris, 2015 | LAMP was not used as the index test and PCR was not used as the reference test/standard | Morris U, Khamis M, Aydin-Schmidt B, Abass A, Msellem M, Nassor M, et al. Field deployment of loop-mediated isothermal amplification for centralized mass-screening of asymptomatic malaria in Zanzibar: A pre-elimination setting. Malaria J. 2015;14:205 |
| 14 | Chen, 2010 | PCR was not used as the reference test/standard | Chen J, Lu F, Lim C, Kim J, Ahn H, Suh I, et al. Detection of *Plasmodium vivax* infection in the Republic of Korea by loop-mediated isothermal amplification (LAMP). Acta Trop 2010;113(1):61-5. |
| 15 | Sirichaisinthop, 2011 | PCR was not used as the reference test/standard | Sirichaisinthop J, Watanabe R, Buates S, Tsuboi T, Takeo S, Krasaesub S, et al. Evaluation of loop-mediated isothermal amplification (LAMP) for malaria diagnosis in a field setting. Am J Trop Med Hyg 2011;85(4):594-6. |
| 16 | Cuadros, 2017 | Samples were not from patients living in malaria endemic areas | Cuadros J, Martin Ramírez A, González I, Ding X, Perez Tanoira R, Rojo-Marcos G, et al. LAMP kit for diagnosis of non-falciparum malaria in *Plasmodium ovale* infected patients. Malaria J. 2017;16:20 |
| 17 | Perera, 2017 | Samples were not from patients living in malaria endemic areas | Perera R, Ding X, Tully F, Oliver J, Bright N, Bell D, et al. Development and clinical performance of high throughput loop-mediated isothermal amplification for detection of malaria. PLoS One. 2017;12(2):e0171126. |
| 18 | Polley, 2010 | Samples were not from patients living in malaria endemic areas | Polley S, Mori Y, Watson J, Perkins M, Gonzalez I, Notomi T, et al. Mitochondrial DNA targets increase sensitivity of malaria detection using loop-mediated isothermal amplification. J Clin Microbiol. 2010;48(8):2866-71 |
| 19 | Tegegne, 2018 | Samples were taking from pregnant women | Tegegne B, Getie S, Lemma W, Mohon AN, Pillai DR. Performance of loop-mediated isothermal amplification (LAMP) for the diagnosis of malaria among malaria suspected pregnant women in Northwest Ethiopia. Malar J. 2017;16(1):34. |
| 20 | Vásquez, 2018 | Samples were taking from pregnant women | Vásquez AM, Zuluaga L, Tobón A, Posada M, Vélez G, González IJ, et al. Diagnostic accuracy of loop-mediated isothermal amplification (LAMP) for screening malaria in peripheral and placental blood samples from pregnant women  in Colombia. Malar J. 2018;17(1):262. |
| 21 | Cheaveau,2018 | Samples were taking from travellers | Cheaveau J, Nguyen H, Chow B, Marasinghe D, Mohon AN, Yuan H, et al. clinical validation of a commercial LAMP test for ruling out malaria in returning travelers: a prospective diagnostic  trial. Open Forum Infect Dis. 2018 Oct 12;5(11):ofy260. doi: 10.1093/ofid/ofy260.  eCollection 2018 Nov. |
| 22 | Viana,2018 | Insufficient data to construct 2x2 diagnostic table | Viana GMR, Silva-Flannery L, Lima Barbosa DR, Lucchi N, do Valle SCN, Farias  S, et al Field evaluation of a real time loop-mediated isothermal amplification assay (RealAmp) for malaria diagnosis in Cruzeiro do Sul, Acre, Brazil. PLoS One. 2018 ;13(7):e0200492. |
