# Supplementary material for: Loop-mediated isothermal amplification (LAMP) test for diagnosis of uncomplicated malaria in endemic areas: a meta-analysis of diagnostic test accuracy
Source: Malar J. 2020 Jun 19;19:211. doi: 10.1186/s12936-020-03283-9 (PMC7305603; doi:10.1186/s12936-020-03283-9)
Supplement: Supplementary file 3 — Additional file 3. Characteristics of included studies. [file 12936_2020_3283_MOESM3_ESM.doc]

Additional File 3; Table S3. Characteristics of included studies

| **Author** | **Ref no** | **Publication year** | **Study design** | **Country** | **Index test** | **Ref test** | **TP** | **FP** | **FN** | **TN** | **Samp size** | **Study year** | **Median age** | **%male** |
| --- | --- | --- | --- | --- | --- | --- | --- | --- | --- | --- | --- | --- | --- | --- |
| Cook | 6 | 2015 | CS | Tanzania | Pan LAMP | qPCR | 15 | 3 | 3 | 975 | 996 | 2013 | 12 | 46 |
| Chutipongvivate | 7 | 2014 | CS | Thailand | LAMP (*Pf*) | nPCR | 70 | 0 | 1 | 33 | 104 | 2014 | NA | NA |
| Hayashida | 8 | 2017 | CS | Zambia | Non *Pf* CZC LAMP (Pan) | nPCR | 8 | 0 | 0 | 27 | 35 | 2013 | NA | NA |
| Hayashida | 8 | 2017 | CS | Zambia | *Pf* CZC LAMP | nPCR | 22 | 0 | 0 | 13 | 35 | 2013 | NA | NA |
| Hayashida | 8 | 2017 | CS | Zambia | Non *Pf* CZC LAMP (Pan) | nPCR | 27 | 0 | 3 | 31 | 61 | 2013 | NA | NA |
| Hayashida | 8 | 2017 | CS | Zambia | *Pf* CZC LAMP | nPCR | 29 | 1 | 1 | 30 | 61 | 2013 | NA | NA |
| Poon | 16 | 2006 | CC | Thailand | *Pf* LAMP | PCR | 96 | 1 | 5 | 100 | 202 | NA | NA | NA |
| Buates | 17 | 2010 | CC | Thailand | Pfs 16 RT-LAMP (*Pf*) | RT-PCR | 30 | 1 | 0 | 51 | 82 | NA | NA | NA |
| Buates | 17 | 2010 | CC | Thailand | Pfs 25 RT-LAMP (*Pf*) | RT-PCR | 15 | 0 | 0 | 67 | 82 | NA | NA | NA |
| Lucchi | 18 | 2010 | CC | Tanzania | Real  Amp LAMP (*Pf*) | nPCR | 90 | 0 | 1 | 15 | 106 | 2004-2006 | NA | NA |
| Lee | 19 | 2012 | CS | Republic of Sao Tome & principe | LAMP (before treatment) (Pan) | PCR | 64 | 1 | 0 | 63 | 128 | 2009 | NA | NA |
| Hopkins | 20 | 2013 | CS | Uganda | PURE LAMP (Pan) | nPCR | 178 | 3 | 21 | 70 | 272 | 2010-2011 | 23 | 36 |
| Hopkins | 20 | 2013 | CS | Uganda | PURE LAMP (Pan) | nPCR | 179 | 11 | 20 | 62 | 272 | 2010-2011 | 23 | 36 |
| Patel | 21 | 2013 | CC | Venezuela | Real  Amp LAMP (*Pv*) | nPCR | 70 | 0 | 4 | 46 | 120 | NA | NA | NA |
| Aydin-Schmidt | 22 | 2014 | CS | Tanzania | Pan LAMP | nPCR | 150 | 6 | 15 | 1159 | 1330 | 2010-2011 | NA | 41 |
| Aydin-Schmidt | 22 | 2014 | CS | Tanzania | *Pf* LAMP | nPCR | 149 | 3 | 16 | 1162 | 1330 | 2010-2011 | NA | 41 |
| Dinzouna-Boutamba | 23 | 2014 | CS | Republic of  Korea | a-tubulin LAMP (*Pv*) | nPCR | 128 | 9 | 0 | 40 | 177 | 2011 | NA | 100 |
| Mohon | 24 | 2014 | CC | Bangladesh | LAMP (*Pf*) | qPCR | 106 | 0 | 2 | 103 | 211 | 2009-2010 | NA | NA |
| Patel | 25 | 2014 | CS | India | RealAmp LAMP (Pan) | nPCR | 91 | 0 | 5 | 45 | 141 | 2010-2012 | 8 | 62 |
| Patel | 25 | 2014 | CS | Thailand | Real  Amp LAMP (Pan) | nPCR | 7 | 4 | 0 | 116 | 127 | 2012 | 24 | 52 |
| Britton | 26 | 2015 | CC | Gambia, Papua New Guinea, Malaysia | HtLAMP-Pg (Pan) | mPCR, nPCR | 260 | 3 | 6 | 15 | 284 | NA | NA | NA |
| Britton | 26 | 2015 | CC | Gambia, Papua New Guinea, Malaysia | HtLAMP (*Pf*) | mPCR, nPCR | 124 | 6 | 4 | 150 | 284 | NA | NA | NA |
| Oriero | 27 | 2015 | CS | Gambia | *Pf* LAMP | PCR | 120 | 7 | 11 | 203 | 341 | 2014 | 9 | 41 |
| Sema | 28 | 2015 | CS | Ethiopia | NINA-LAMP (Pan) | nPCR | 30 | 8 | 1 | 43 | 82 | 2014 | NA | NA |
| Britton | 29 | 2016 | CC | Malaysia | HtLAMP (*Pk*) | mPCR, nPCR | 25 | 14 | 1 | 6 | 46 | 2014 | NA | NA |
| Britton | 30 | 2016 | CC | Malaysia | HtLAMP (*Pv*) | mPCR, nPCR | 67 | 43 | 5 | 142 | 257 | 2012 | NA | NA |
| Lucchi | 31 | 2016 | CS | Senegal | GFP-LAMP (Pan) | qPCR | 140 | 8 | 4 | 57 | 209 | 2015 | NA | 61 |
| Lucchi | 31 | 2016 | CS | Senegal | SFP-LAMP (Pan) | qPCR | 140 | 4 | 4 | 61 | 209 | 2015 | NA | 61 |
| Kemleu | 32 | 2016 | CC | Cameroon | RT-LAMP (RNA extracts) (*Pf*) | RT-PCR | 106 | 13 | 4 | 9 | 132 | NA | NA | NA |
| Kemleu | 32 | 2016 | CC | Cameroon | RT-LAMP (Whole blood) (*Pf*) | RT-PCR | 99 | 13 | 11 | 9 | 132 | NA | NA | NA |
| Ocker | 33 | 2016 | CC | Thailand | Pan LAMP | nPCR | 116 | 0 | 1 | 33 | 150 | 2011 | NA | NA |
| Ocker | 33 | 2016 | CC | Thailand | *Pf* LAMP | nPCR | 73 | 4 | 4 | 69 | 150 | 2011 | NA | NA |
| Aydin-Schmidt | 34 | 2017 | CS | Tanzania | HTP-LAMP (Pan) | qPCR | 20 | 2 | 29 | 2957 | 3008 | 2015 | 14 | 41 |
| Piera | 35 | 2017 | CC | Malaysia | Pan LAMP | PCR | 73 | 0 | 0 | 19 | 92 | 2012-2014 | NA | NA |
| Piera | 35 | 2017 | CC | Malaysia | *Pf* LAMP | PCR | 20 | 0 | 0 | 72 | 92 | 2012-2014 | NA | NA |
| Serra-Casas | 36 | 2017 | CS | Peru | Pan LAMP | qPCR | 235 | 20 | 21 | 227 | 503 | 2013 | 22 | 46 |
| Singh | 37 | 2017 | CS | India | Pan LAMP | nPCR | 24 | 0 | 11 | 58 | 317 | 2011-20183 | NA | 70 |
| Singh | 37 | 2017 | CS | India | *Pf* LAMP | nPCR | 134 | 0 | 3 | 180 | 317 | 2011-2013 | NA | 70 |
| Singh | 37 | 2017 | CS | India | *Pv* LAMP | nPCR | 124 | 0 | 8 | 185 | 317 | 2011-2013 | NA | 70 |
| Kaur | 38 | 2018 | CC | India | *Pv* LAMP | RT-PCR | 148 | 0 | 0 | 17 | 165 | 2013-2016 | NA | NA |
| Kudyba | 39 | 2019 | CC | Brazil | MG-LAMP (Pan) | PCR | 36 | 3 | 4 | 48 | 91 | 2017 | NA | NA |
| Kudyba | 39 | 2019 | CC | Brazil | MG-LAMP (*Pf*) | PCR | 10 | 0 | 2 | 27 | 39 | 2017 | NA | NA |
| Kudyba | 39 | 2019 | CC | Brazil | MG-LAMP (*Pv*) | PCR | 25 | 0 | 2 | 12 | 39 | 2017 | NA | NA |
