# Supplementary material for: Loop-mediated isothermal amplification (LAMP) test for diagnosis of uncomplicated malaria in endemic areas: a meta-analysis of diagnostic test accuracy
Source: Malar J. 2020 Jun 19;19:211. doi: 10.1186/s12936-020-03283-9 (PMC7305603; doi:10.1186/s12936-020-03283-9)
Supplement: Supplementary file 4 — Additional file 4. Summary of the methodological quality assessment across all studies. [file 12936_2020_3283_MOESM4_ESM.doc]

Additional File 4: Table S4. Test performance of individual studies with Pan LAMP

| **Author, yr** | **Ref no.** | **samples** | **TP** | **FP** | **TN** | **FN** |
| --- | --- | --- | --- | --- | --- | --- |
| Cook,  2015 | 6 | 996 | 15 | 3 | 975 | 3 |
| Hayashida,2017 | 8 | 35 | 8 | 0 | 27 | 0 |
| Hayashida,2017 | 8 | 61 | 27 | 0 | 31 | 3 |
| Lee, 2012 | 19 | 128 | 64 | 1 | 63 | 0 |
| Hopkins,  2013 | 20 | 272 | 178 | 3 | 70 | 21 |
| Hopkins,  2013 | 20 | 272 | 179 | 11 | 62 | 20 |
| Aydin-Schmidt, 2014 | 22 | 1,330 | 150 | 6 | 1159 | 15 |
| Patel,2014 | 25 | 141 | 91 | 0 | 45 | 5 |
| Patel,2014 | 25 | 127 | 7 | 4 | 116 | 0 |
| Sema,2015 | 28 | 82 | 30 | 8 | 43 | 1 |
| Lucchi,2016 | 31 | 209 | 140 | 8 | 57 | 4 |
| Lucchi,2016 | 31 | 209 | 140 | 4 | 61 | 4 |
| Ocker,2016 | 33 | 150 | 116 | 0 | 33 | 1 |
| Aydin-Schmidt,  2017 | 34 | 3,008 | 20 | 2 | 2957 | 29 |
| Piera,2017 | 35 | 92 | 73 | 0 | 19 | 0 |
| Kudyba,2019 | 39 | 91 | 36 | 3 | 48 | 4 |

TP: true positive; FP: false positive; TN: true negative; FN: false negative
