# Supplementary figures and images for: Loop-mediated isothermal amplification (LAMP) test for diagnosis of uncomplicated malaria in endemic areas: a meta-analysis of diagnostic test accuracy
Source: Malar J. 2020 Jun 19;19:211. doi: 10.1186/s12936-020-03283-9 (PMC7305603; doi:10.1186/s12936-020-03283-9)

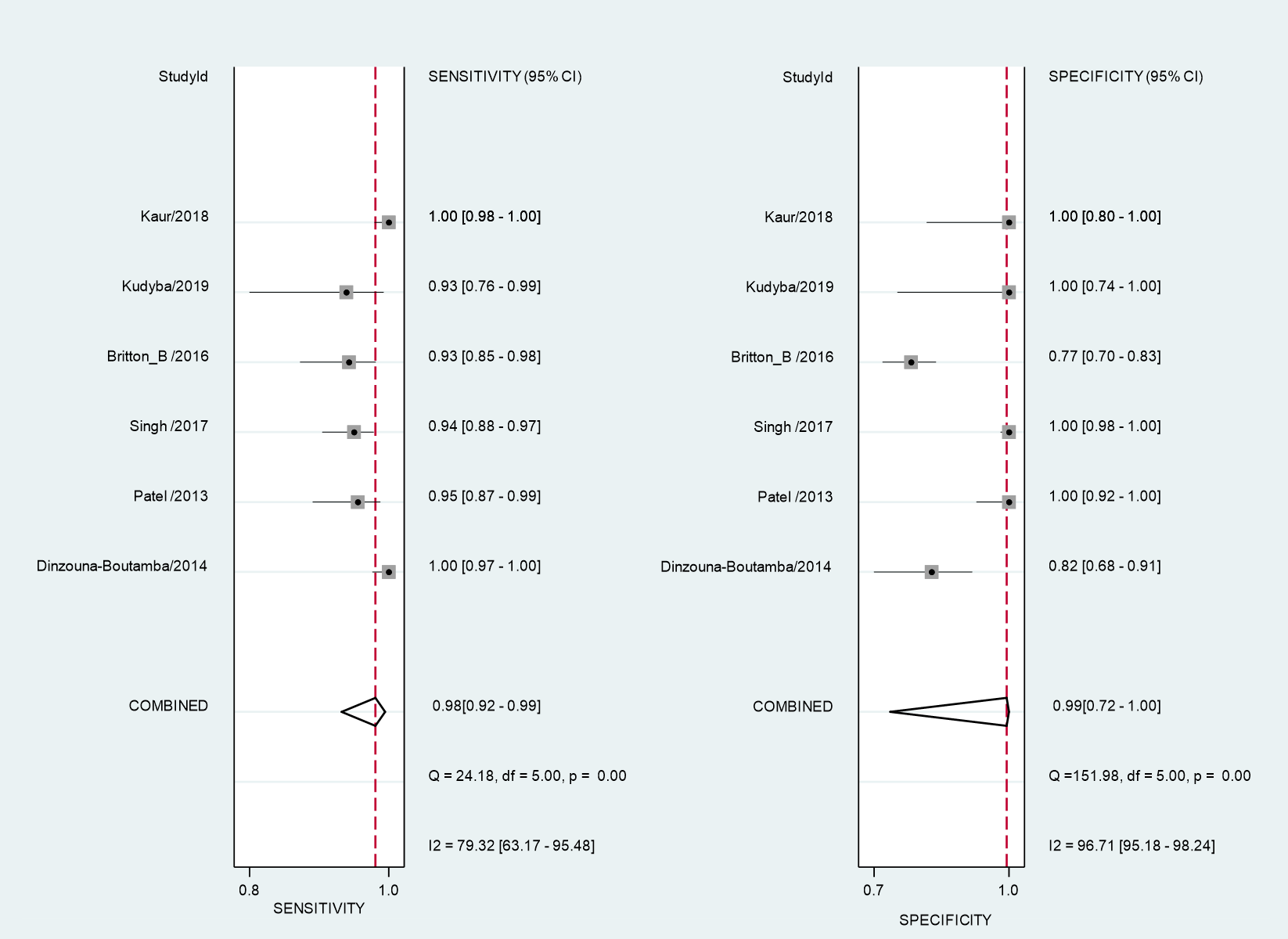

Supplement: Supplementary file 6 — Additional file 6. Forest plot of sensitivity and specificity for Pv LAMP. [file 12936_2020_3283_MOESM6_ESM.tiff]

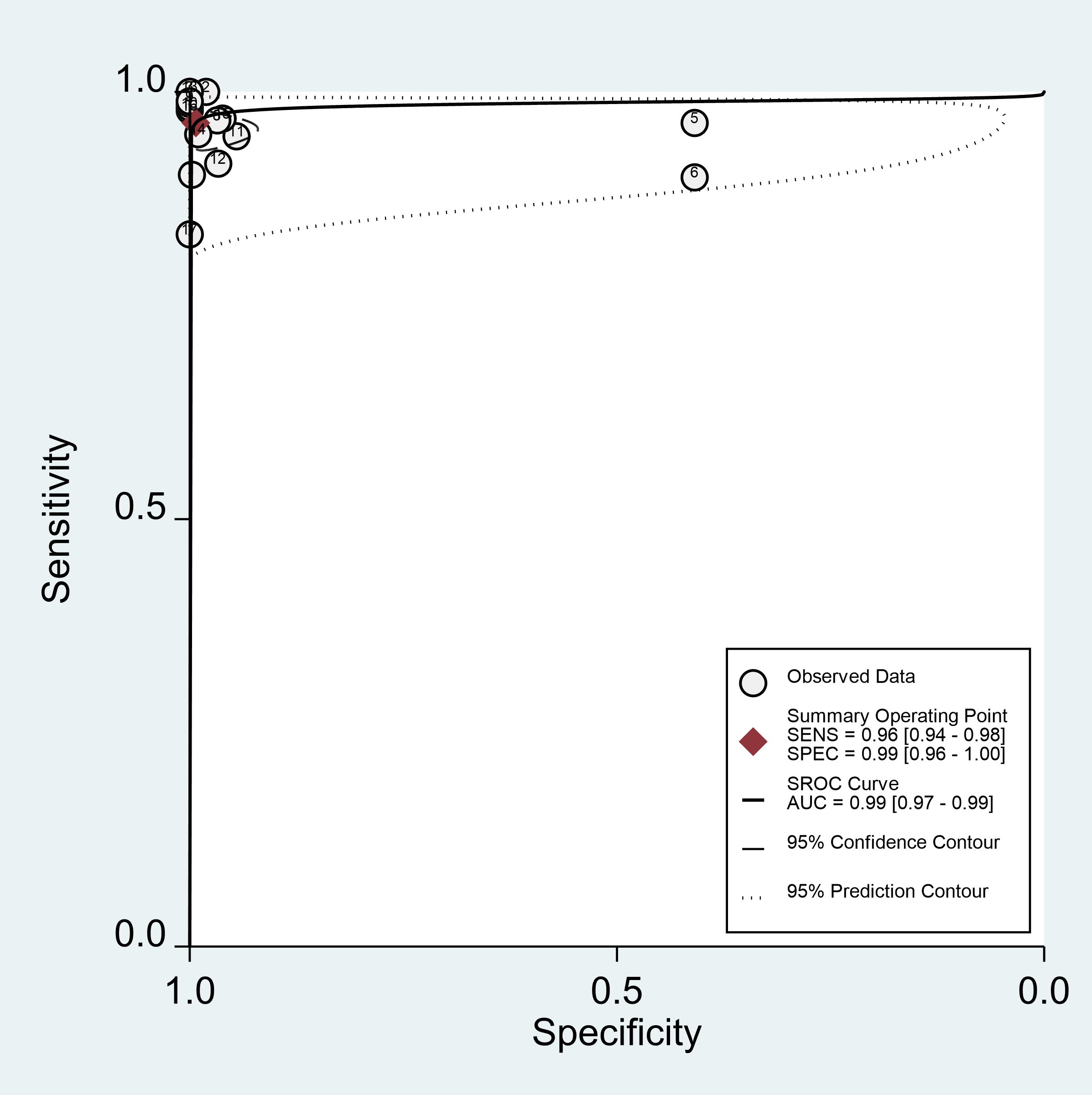

Supplement: Supplementary file 7 — Additional file 7. SROC plot of studies that used Pf LAMP. [file 12936_2020_3283_MOESM7_ESM.tiff]

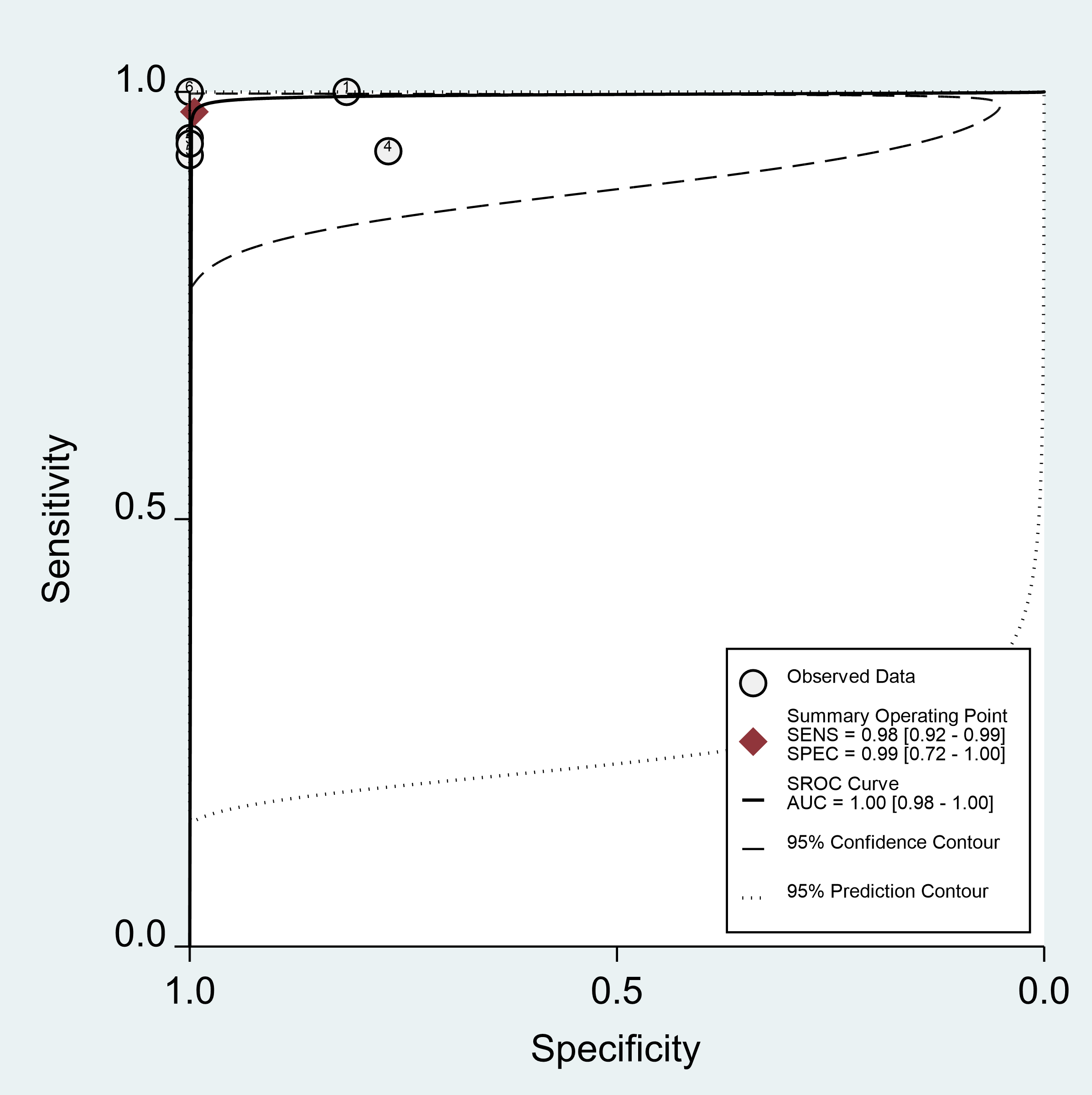

Supplement: Supplementary file 8 — Additional file 8. SROC plot of studies that used Pv LAMP. [file 12936_2020_3283_MOESM8_ESM.tiff]
